# Circulating Malondialdehyde-Modified LDL-Related Variables and Coronary Artery Stenosis in Asymptomatic Patients with Type 2 Diabetes

**DOI:** 10.1155/2015/507245

**Published:** 2015-03-26

**Authors:** Kazuya Fujihara, Hiroaki Suzuki, Akira Sato, Satoru Kodama, Yoriko Heianza, Kazumi Saito, Hitoshi Iwasaki, Kazuto Kobayashi, Shigeru Yatoh, Akimitsu Takahashi, Naoya Yahagi, Hiroaki Yagyu, Hirohito Sone, Hitoshi Shimano

**Affiliations:** ^1^Division of Endocrinology and Metabolism, Department of Internal Medicine, Faculty of Medicine, University of Tsukuba, Tsukuba, Ibaraki 305-8575, Japan; ^2^Division of Cardiology, Department of Internal Medicine, Faculty of Medicine, University of Tsukuba, Tsukuba, Ibaraki 305-8575, Japan; ^3^Department of Hematology, Endocrinology and Metabolism, School of Medicine, Niigata University, Niigata, Niigata 951-8510, Japan; ^4^Division of Endocrinology and Metabolism, Center for Medical Sciences, Ibaraki Prefectural University of Health Sciences, Ami, Ibaraki 300-0331, Japan; ^5^International Institute for Integrative Sleep Medicine (WPI-IIIS), University of Tsukuba, Tsukuba, Ibaraki 305-8575, Japan

## Abstract

*Aims*. To elucidate the levels of malondialdehyde-modified LDL (MDA-LDL)-related variables for predicting coronary artery stenosis (CAS) by coronary CT angiography (CCTA) in asymptomatic patients with type 2 diabetes (T2DM).* Methods*. Enrolled were 36 Japanese patients with T2DM who underwent CCTA and in whom MDA-LDL levels were measured. Definition of CAS was luminal narrowing of ≥50%. Trends through tertiles of each MDA-LDL-related variable were analyzed with a general linear model. The ability of each MDA-LDL-related variable to predict CAS was compared to areas under the curve (AUCs) in receiver operating characteristic curve (ROC) analysis.* Results*. Seventeen patients had CAS. Each MDA-LDL-related variable was an independent predictor of CAS (*P* = 0.039 for MDALDL, *P* = 0.013 for MDA-LDL/LDL-C, *P* = 0.047 for MDA-LDL/HDL-C, and *P* = 0.013 for (MDA-LDL/LDL-C)/HDL-C). AUCs of MDA-LDL, MDA-LDL/LDL-C, MDA-LDL/HDL-C, and (MDA-LDL/LDL-C)/HDL-C were 0.675 (95% CI 0.496–0.854), 0.765 (0.602–0.927), 0.752 (0.592–0.913), and 0.799 (0.643–0.955), respectively, for predicting CAS. Trends throughout the tertiles showed significant associations between MDA-LDL/LDL-C, MDA-LDL/HDL-C, or (MDALDL/LDL-C)/HDL-C and CAS (*P* = 0.003 for MDA-LDL/LDL-C, *P* = 0.042 for MDA-LDL/HDL-C, and *P* = 0.001 for (MDA-LDL/LDL-C)/HDL-C).* Conclusions*. Data suggest that measurements of MDA-LDL/LDL-C, MDA-LDL/HDLC, and (MDA-LDL/LDL-C)/HDL-C are useful for predicting CAS.

## 1. Introduction

Coronary artery disease (CAD) is a common cause of mortality in patients with type 2 diabetes [1]. Although the conventional risk factors for CAD among adults with diabetes have improved significantly in recent years [2], a considerable number of patients still develop CAD even under intensive management. Prospective observational studies using coronary CT angiography (CCTA) showed that obstructive CAD precedes coronary events [3, 4]. Therefore, identifying patients with coronary artery stenosis (CAS) is important in implementing appropriate management to prevent coronary events in asymptomatic patients with type 2 diabetes.

Oxidized LDL induces differentiation of monocytes into macrophages and proliferation of vascular smooth muscle cell stimulation, resulting in increased foam cell formation in atherosclerotic lesions, endothelial injury, and plaque formation [5, 6]. Malondialdehyde-modified low-density lipoprotein (MDA-LDL), known as an oxidized LDL, could play key roles in the progression of atherosclerosis [5, 7]. Previous studies showed that increased serum MDA-LDL levels were associated with CAD [8–10] or coronary artery calcification [11]. Serum MDA-LDL levels have been positively correlated with carotid intima-media thickness [9, 12]. Moreover, it was shown that the MDA-LDL-to-LDL-cholesterol (LDL-C) ratio (MDA-LDL/LDL-C) was a more useful predictor of coronary artery calcification than the MDA-LDL level alone [11, 13]. Kurobe et al. revealed that the vascular protective effects of ezetimibe, an inhibitor of cholesterol intestinal absorption, were correlated with decreased values of MDA-LDL and MDA-LDL/LDL-C but not with those of LDL-C [14]. In that study, those effects were more closely correlated with the reduction of the MDA-LDL/LDL-C compared with that of MDA-LDL [14].

A low serum level of high-density lipoprotein cholesterol (HDL-C) is an independent risk factor for CAD in type 2 diabetes [15]. We and other groups showed that HDL-C was significantly lower in patients with CAS detected by CCTA than in those without CAS [16–18]. The antiatherogenic property of HDL-C is caused by promotion of reverse cholesterol transport and the antioxidant ability of HDL-C [19].

The total-to-HDL cholesterol ratio and LDL-to-HDL-C (LDL-C/HDL-C) ratio were reported to be better predictors of future cardiovascular disease [20] and CAS [16] than single lipid parameters. Therefore, we hypothesized that the use of MDA-LDL and MDA-LDL-related variables, including MDA-LDL/LDL-C, MDA-LDL-to-HDL-C ratio (MDA-LDL/HDL-C), and (MDA-LDL/LDL-C)-to-HDL-C ratio (MDA-LDL/LDL-C)/HDL-C, in predicting CAS would help to effectively identify patients at risk for CAS. This study aimed to elucidate the levels of these MDA-LDL-related variables for predicting CAS by 64-slice CCTA in asymptomatic patients with type 2 diabetes.

## 2. Methods

### 2.1. Subjects

We retrospectively analyzed data on patients with type 2 diabetes who underwent CCTA and measurements of MDA-LDL at the University of Tsukuba Hospital from April 2009 to March 2012. Because of complications associated with CCTA, such as renal failure, allergy, and radiation-related issues, we reserve its use for those patients at high risk for CAD in whom the risk/benefit ratio indicates its use. Reasons for performing CCTA and exclusion criteria were described elsewhere [16, 17]. All patients had undergone a structured interview, physical examination, and laboratory analysis. Hypertension was defined as systolic blood pressure ≥140 mmHg and/or diastolic blood pressure ≥90 mmHg or the current use of antihypertensive agents. This study was approved by the institutional ethics committee and conducted according to the Helsinki Declaration.

### 2.2. Laboratory Analysis

Blood samples were collected the morning after an overnight fast. Plasma levels of glucose and serum levels of total cholesterol, HDL-C, triglycerides, and creatinine were determined by an automated analyzer (7700 clinical analyzer; Hitachi High-Technologies Corporation, Tokyo, Japan). Serum LDL-C levels were calculated by the Friedewald equation. HbA1c was measured by high-performance liquid chromatography (HLC-723G9; Tosoh Corporation, Tokyo, Japan). Serum MDA-LDL levels were measured by a sandwich enzyme-linked immunosorbent assay (Sekisui Medical, Tokyo, Japan). HbA1c values were converted from the Japanese Diabetes Society values to National Glycohemoglobin Standardization Program equivalent values [21].

### 2.3. Assessment of CCTA

Coronary stenosis was assessed with a Philips Brilliance-64 scanner (Philips Medical Systems, Cleveland, OH, USA) with a 64 × 0.625-mm detector configuration. Scanning and data analysis were performed as described previously [16, 17]. Luminal narrowing of ≥50% on CCTA was defined as CAS.

### 2.4. Statistical Analysis

Categorical variables were expressed as numerals and percentages and were compared with Fisher's exact tests. Continuous variables were expressed as mean ± SD or median and interquartile range. Based on distribution, continuous variables were compared using unpaired Student's *t*-tests or Mann-Whitney *U* tests. Based on the degrees of CAS, MDA-LDL-related variables were compared using one-way ANOVA. Logistic regression analyses identified variables related to CAS. Differences across tertiles of the MDA-LDL-related variables were analyzed with a general linear model. Differences across tertiles of each MDA-LDL-related variable were analyzed with one-way ANOVA followed by the Bonferroni* post hoc* test. The ability of each MDA-LDL-related variable to predict CAS was compared to areas under the curve (AUCs) in receiver operating characteristic curve (ROC) analysis. All statistical analyses were performed by SPSS (version 18.0, Chicago, IL). Statistical significance was considered for *P* < 0.05.

## 3. Results

### 3.1. Characteristics of Subjects

Initially enrolled were 37 patients. However, 1 patient was excluded because of the presence of hypertriglyceridemia.  Table 1 lists subjects' baseline characteristics according to the presence of CAS. As shown in Table 1, compared with those without stenosis (stenosis [−] group), more patients with stenosis (stenosis [+] group) had a longer duration of diabetes, higher systolic blood pressure, higher rate of retinopathy, higher MDA-LDL/LDL-C, or higher (MDA-LDL/LDL-C)/HDL-C; differences in these parameters between the stenosis (+) and stenosis (−) groups were significant. The HDL-C level, however, was significantly lower in the stenosis (+) group than in the stenosis (−) group (Table 1).  Table 2 shows the comparison of each MDA-LDL-related variable according to the degrees of CAS. The values for MDA-LDL/LDL-C or (MDA-LDL/LDL-C)/HDL-C were significantly different among the four groups.

### 3.2. Logistic Regression Analyses for Prediction of CAS

Table 3 shows logistic regression analyses for prediction of CAS for each MDA-LDL-related variable. MDA-LDL (odds ratio (OR) 1.02 (95% confidence interval 1.00–1.04), *P* = 0.039), MDA-LDL/LDL-C (1.13 (1.03–1.25), *P* = 0.013), MDA-LDL/HDL-C (1.02 (1.00–1.05), *P* = 0.047), and (MDA-LDL/LDL)/HDL-C (1.16 (1.03–1.30), *P* = 0.013) were independent predictors of CAS after adjustments for age, sex, body mass index, hypertension, duration of diabetes, smoking, and HbA1c.

### 3.3. Each MDA-LDL-Related Variable and Presence of CAS

**Figure 1:**
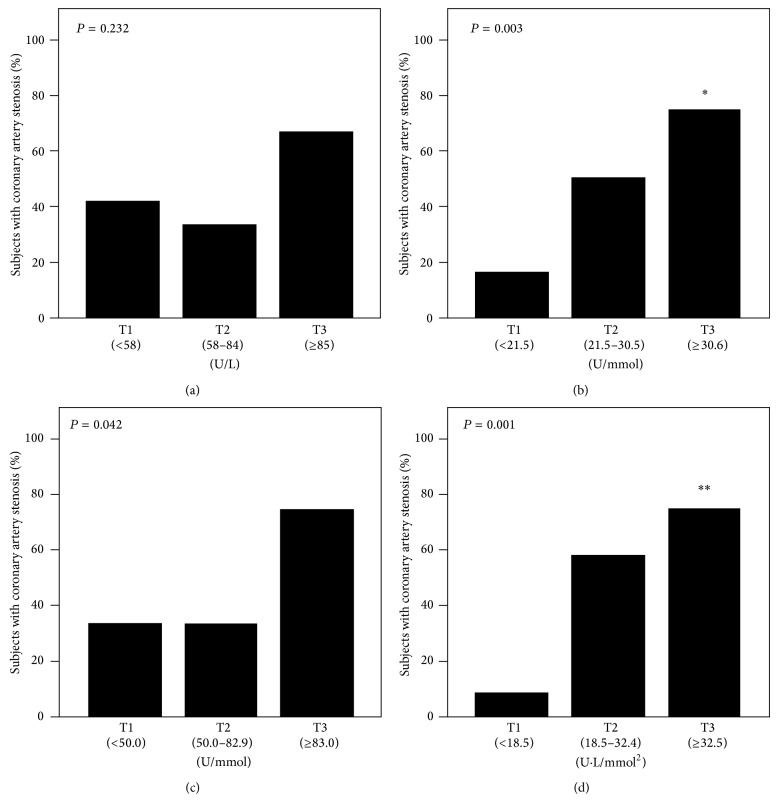
Percentage of subjects with coronary artery stenosis in tertiles (T) of (a) MDA-LDL, (b) MDA-LDL/LDL-C, (c) MDA-LDL/HDL-C, and (d) (MDA-LDL/LDL-C)/HDL-C. There was a significant association between MDA-LDL/LDL-C, MDA-LDL/HDL-C or (MDA-LDL/LDL-C)/HDL-C and the presence of coronary artery stenosis (*P* = 0.003 for MDA-LDL/LDL-C, *P* = 0.042 for MDA-LDL/HDL-C, *P* = 0.001 for (MDA-LDL/LDL-C)/HDL-C). MDA-LDL, malondialdehyde-modified LDL; MDA-LDL/LDL-C, MDA-LDL-to-LDL cholesterol ratio; MDA-LDL-C/HDL-C, MDA-LDL-to-HDL cholesterol ratio. ^∗^ *P* < 0.05versuss T1 and ^∗∗^ *P* < 0.01.

Figure 1 shows the results of tertile analysis of each MDA-LDL-related variable for the presence of CAS. According to MDA-LDL tertiles, 5 patients (42%) in the lowest tertile (T1), 4 (33%) in the middle tertile (T2), and 8 (67%) in the highest tertile (T3) had CAS (Figure 1(a)). CAS was observed in 2 patients (17%) in T1, 6 (50%) in T2, and 9 (75%) in T3 according to MDA-LDL/LDL-C tertiles (Figure 1(b)). Four (33%) had CAS in T1, 4 (33%) in T2, and 9 (75%) in T3 according to MDA-LDL/HDL-C tertiles (Figure 1(c)). CAS was observed in 1 patient (8%) in T1, 7 patients (58%) in T2, and 9 patients (75%) in T3 according to (MDA-LDL/LDL-C)/HDL-C tertiles (Figure 1(d)). Analyses of trends throughout the tertiles of MDA-LDL/LDL-C, MDA-LDL/HDL-C, and (MDA-LDL/LDL-C)/HDL-C showed significant associations between those variables and the presence of CAS (*P* = 0.003 for MDA-LDL/LDL-C, *P* = 0.042 for MDA-LDL/HDL-C, and *P* = 0.001 for (MDA-LDL/LDL-C)/HDL-C). Statistical significance was observed between T1 and T3 in MDA-LDL/LDL-C (*P* = 0.011) and (MDA-LDL/LDL-C)/HDL-C (*P* = 0.002).

### 3.4. AUCs of Each MDA-LDL-Related Variable for the Prediction of CAS

**Figure 2:**
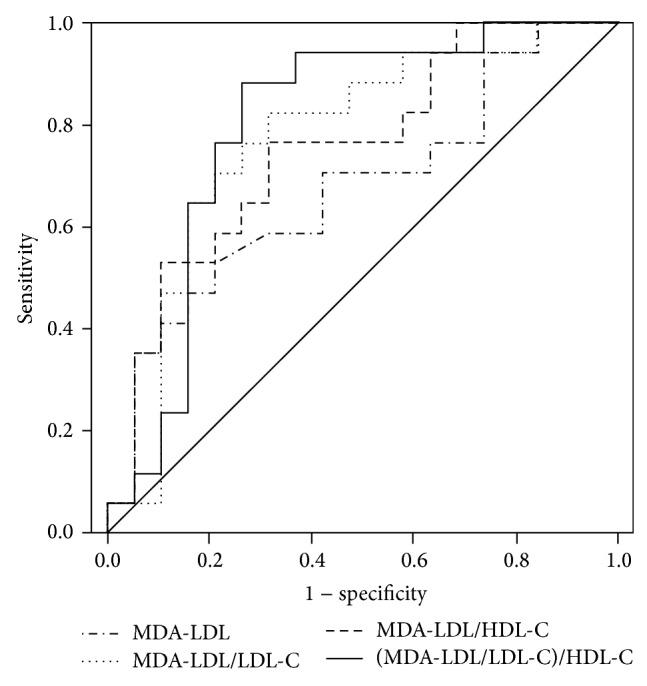
Comparison among the AUCs of MDA-LDL, MDA-LDL/LDL-C, MDA-LDL/HDL-C, and (MDA-LDL/LDL-C)/HDL-C for the presence of coronary artery stenosis. The AUCs (95% confidence interval) were as follows: MDA-LDL 0.675 (0.496–0.854), MDA-LDL/LDL-C 0.765 (0.602–0.927), MDA-LDL/HDL-C 0.752 (0.592–0.913), and (MDA-LDL/LDL-C)/HDL-C 0.799 (0.643–0.955). MDA-LDL: malondialdehyde-modified LDL; MDA-LDL/LDL-C: MDA-LDL-to-LDL cholesterol ratio; MDA-LDL-C/HDL-C: MDA-LDL-to-HDL cholesterol ratio; (MDA-LDL/LDL-C)/HDL-C: (MDA-LDL/LDL-C) to the HDL cholesterol ratio.

The AUCs in ROC curve analyses of each MDA-LDL-related variable are shown in Table 4 and Figure 2. MDA-LDL/LDL-C, MDA-LDL/HDL-C, and (MDA-LDL/LDL-C)/HDL-C showed significant discriminative ability for CAS.

## 4. Discussion

The present results showed by logistic regression analyses that MDA-LDL-related variables as well as MDA-LDL levels were independent predictors of CAS in asymptomatic type 2 diabetic patients. MDA-LDL/LDL-C, MDA-LDL/HDL-C, or (MDA-LDL/LDL-C)/HDL-C were significantly associated with the presence of CAS in the analyses of trends throughout the tertiles. In the ROC analyses, not MDA-LDL alone but only the MDA-LDL/LDL-C, MDA-LDL/HDL-C, and (MDA-LDL/LDL-C)/HDL-C variations could predict CAS.

Although increased serum levels of MDA-LDL are associated with the presence of CAD [8–10], several studies showed that MDA-LDL/LDL-C was more useful than MDA-LDL alone [11, 13]. Our results were consistent with these results. Serum MDA-LDL levels were significantly correlated with serum levels of LDL-C [9] or small dense LDL [22], which is susceptible to lipid peroxidation [23]. Moreover, serum levels of MDA-LDL were higher in diabetic patients compared with nondiabetic individuals with the same LDL size ranges [22]. Therefore, MDA-LDL/LDL-C might indicate the degree of oxidative stress in patients with type 2 diabetes. Interestingly, in the current study, the levels of LDL-C were lower in patients with CAS compared to those without CAS, suggesting that oxidative stress is more important for CAS than LDL-C in patients with type 2 diabetes at high risk of CAD.

The ratio of an atherogenic risk variable to an antiatherogenic variable could be a better predictor of coronary heart disease (CHD) compared with an atherogenic or antiatherogenic risk variable alone. Indeed, the LDL-C/HDL-C ratio is a better predictor of CHD [20], cardiovascular disease [24], or carotid intima-media thickness progression [25] than HDL-C or LDL-C alone. Moreover, changes in the LDL-C/HDL-C ratio were shown to be a better indicator of successful CHD risk reduction than changes in absolute levels of LDL-C [26]. We previously reported that LDL-C/HDL-C was an independent predictor of CAS [16]. A similar result was reported by Hamsten et al. in that LDL-C/HDL-C was significantly associated with the atheromatosis score [27]. However, the AUC in the ROC for the prediction of CAS or the correlation coefficient between LDL-C/HDL-C and the atheromatosis score was not very high (AUC = 0.618, *r* = 0.472) [16, 24]. In the current study, the AUC for the prediction of CAS was higher for MDA-LDL/HDL-C than for MDA-LDL alone in ROC analyses; similarly, the AUC was higher for (MDA-LDL/LDL-C)/HDL-C than for MDA-LDL/LDL-C. These data suggest that the ratio of MDA-LDL or MDA-LDL/LDL-C to HDL-C is more useful as a predictor of CAS compared with MDA-LDL or MDA-LDL/LDL-C in patients with type 2 diabetes at high risk for CAD. However, no significant differences were observed in the values for MDA-LDL/HDL-C in the analysis according to the degrees of CAS. Future studies are needed to clarify the clinical usefulness of MDA-LDL/HDL-C and (MDA-LDL/LDL-C)/HDL-C in the prediction of CAS.

Several limitations should be addressed regarding this study. First, it was cross-sectional with a small number of subjects. The number of patients was too small for a clinically useful ROC analysis in terms of sensitivity and specificity. Our findings could not explain the causality between each MDA-LDL-related variable and the presence of CAS. Therefore, our results should be confirmed in studies with an appropriate sample size that can determine clinically useful cutoff values for CAS. Also, prospective studies are needed to assess the association between MDA-LDL-related variables and the development of future CAD in asymptomatic patients with diabetes. Second, all of our subjects were asymptomatic, but they were at high risk for cardiovascular disease. Future studies are needed to evaluate our findings in patients with type 2 diabetes at low to moderate risk for CAD. Third, our participants had poor glycemic control since all of the subjects had been admitted for the treatment of diabetes.

In conclusion, the measurement of MDA-LDL/LDL-C, MDA-LDL/HDL-C, and (MDA-LDL/LDL-C)/HDL-C may be useful in the prediction of CAS in asymptomatic patients with type 2 diabetes.

## Figures and Tables

**Table 1:** Characteristics of study participants.

	Coronary artery stenosis		*P* value
	(−)	(+)	
	*n* = 19	*n* = 17	
Age (years)	57 ± 15	62 ± 7	0.094
Male/female	9/10	12/5	0.192
BMI (kg/m^2^)	26.4 ± 5.7	27.2 ± 4.8	0.642
Duration of diabetes (years)	3.0 (1.0–11.0)	6.0 (12.0–20.5)	0.007
Hypertension, *n* (%)	12 (63)	14 (82)	0.274
Systolic blood pressure (mmHg)	123 ± 19	138 ± 15	0.015
Diastolic blood pressure (mmHg)	73 ± 13	77 ± 10	0.448
Smoking, *n* (%)	10 (53)	8 (47)	1.000
Retinopathy, *n* (%)	3 (16)	12 (71)	0.002
Nephropathy, *n* (%)	6 (32)	11 (65)	0.057
Neuropathy, *n* (%)	12 (63)	14 (83)	0.274
HbA1c (%)	10.1 ± 1.8	9.5 ± 1.3	0.313
Fasting plasma glucose (mmol/L)	8.1 ± 2.4	7.9 ± 2.0	0.856
Total cholesterol (mmol/L)	5.02 ± 0.96	4.58 ± 0.97	0.183
LDL cholesterol (mmol/L)	3.13 ± 0.82	2.76 ± 0.79	0.185
HDL cholesterol (mmol/L)	1.20 ± 0.38	0.98 ± 0.15	0.031
MDA-LDL (U/L)	70 ± 40	93 ± 57	0.161
MDA-LDL/LDL-C (U/mmol)	23.3 ± 12.3	32.9 ± 11.3	0.020
MDA-LDL/HDL-C (U/mmol)	63.8 ± 45.3	100.3 ± 74.4	0.080
(MDA-LDL/LDL-C)/HDL-C (U·L/mmol^2^)	21.3 ± 13.8	34.9 ± 15.2	0.008
Triglycerides (mmol/L)	1.50 ± 0.60	1.82 ± 0.71	0.148
Medications, *n* (%)			
Insulin treatment	8 (42)	3 (18)	0.156
Sulfonylurea	6 (32)	6 (35)	1.000
Glinides	2 (11)	2 (12)	1.000
Metformin	7 (37)	9 (53)	0.503
Thiazolidinedione	0 (0)	2 (12)	0.216
*α*-Glucosidase inhibitor	0 (0)	3 (18)	0.095
Incretin-related therapies	5 (26)	8 (47)	0.299
Statin	8 (42)	7 (41)	1.000

Data are mean ± SD or median (interquartile range). BMI: body mass index; hypertension: systolic blood pressure ≥140 mmHg and/or diastolic blood pressure ≥90 mmHg or treatment; MDA-LDL: malondialdehyde-modified LDL; MDA-LDL-C/LDL-C: MDA-LDL-to-LDL cholesterol ratio; MDA-LDL-C/HDL-C: MDA-LDL-to-HDL cholesterol ratio; (MDA-LDL/LDL-C)/HDL-C: (MDA-LDL/LDL-C) to HDL cholesterol ratio.

**Table 2:** Comparison of MDA-LDL-related variables according to the degrees of coronary artery stenosis.

	Degrees of coronary artery stenosis				*P* value
	<25%	25–<50%	50–<75%	75%-	
	*n* = 11	*n *= 8	*n* = 5	*n* = 12	
MDA-LDL (U/L)	81 ± 46	55 ± 26	75 ± 29	101 ± 64	0.236
MDA-LDL/LDL-C (U/mmol)	26.3 ± 10.7	19.2 ± 13.7	28.1 ± 11.0	34.9 ± 11.2	0.044
MDA-LDL/HDL-C (U/mmol)	76.4 ± 54.0	46.5 ± 23.0	69.3 ± 24.3	113.2 ± 85.0	0.116
(MDA-LDL/LDL-C)/HDL-C (U·L/mmol^2^)	24.6 ± 13.6	16.9 ± 13.8	26.5 ± 9.5	38.6 ± 15.9	0.013

Data are mean ± SD. MDA-LDL: malondialdehyde-modified LDL; MDA-LDL-C/LDL-C: MDA-LDL-to-LDL cholesterol ratio; MDA-LDL-C/HDL-C: MDA-LDL-to-HDL cholesterol ratio; (MDA-LDL/LDL-C)/HDL-C: (MDA-LDL/LDL-C) to HDL cholesterol ratio.

**Table 3:** Logistic regression models for variables associated with the presence of coronary artery stenosis.

	OR (95% CI)	*P* value	OR (95% CI)	*P* value	OR (95% CI)	*P* value	OR (95% CI)	*P* value
Age	1.13 (1.00–1.28)	0.042	1.16 (1.01–1.32)	0.033	1.14 (1.01–1.29)	0.033	1.18 (1.02–1.37)	0.031
Male sex	3.00 (0.33–27.7)	0.332	3.13 (0.27–36.1)	0.360	3.53 (0.29–42.3)	0.320	4.99 (0.24–104.4)	0.300
BMI	1.16 (0.94–1.44)	0.172	1.13 (0.90–1.43)	0.288	1.15 (0.92–1.44)	0.218	1.10 (0.84–1.43)	0.485
Hypertension	0.28 (0.02–4.11)	0.353	0.17 (0.01–3.28)	0.239	0.24 (0.01–4.95)	0.357	0.16 (0.01–5.06)	0.301
Duration of diabetes	1.17 (1.01–1.35)	0.031	1.16 (0.99–1.36)	0.068	1.20 (1.02–1.42)	0.031	1.23 (0.99–1.54)	0.063
Smoking	0.27 (0.03–2.14)	0.216	0.16 (0.02–1.77)	0.136	0.25 (0.03–2.19)	0.211	0.12 (0.01–1.94)	0.135
HbA1c	1.20 (0.58–2.50)	0.627	1.37 (0.61–3.10)	0.450	1.27 (0.59–2.74)	0.547	1.50 (0.62–3.62)	0.365
MDA-LDL	1.02 (1.00–1.04)	0.039	—	—	—	—	—	—
MDA-LDL/LDL-C (per U/mmol increment)	—	—	1.13 (1.03–1.25)	0.013	—	—	—	—
MDA-LDL/HDL-C (per U/mmol increment)	—	—	—	—	1.02 (1.00–1.05)	0.047	—	—
(MDA-LDL/LDL-C)/HDL-C (per U·L/mmol^2^increment)	—	—	—	—	—	—	1.16 (1.03–1.30)	0.013

BMI: body mass index; hypertension: systolic blood pressure ≥140 mmHg and/or diastolic blood pressure ≥90 mmHg or treatment; MDA-LDL: malondialdehyde-modified LDL; MDA-LDL-C/LDL-C: MDA-LDL-to-LDL cholesterol ratio; MDA-LDL-C/HDL-C: MDA-LDL-to-HDL cholesterol ratio; (MDA-LDL/LDL-C)/HDL-C: (MDA-LDL/LDL-C) to HDL cholesterol ratio.

**Table 4:** Area under the receiver operating characteristic curves for variables associated with the presence of coronary artery stenosis.

	AUCROC (95% CI)	*P* value
MDA-LDL	0.675 (0.496, 0.854)	0.073
MDA-LDL/LDL-C	0.765 (0.602, 0.927)	0.007
MDA-LDL/HDL-C	0.752 (0.592, 0.913)	0.010
(MDA-LDL/LDL-C)/HDL-C	0.799 (0.643, 0.955)	0.002

AUCROC: area under the receiver operating characteristic curve; CI: confidence interval; MDA-LDL: malondialdehyde-modified LDL, MDA-LDL-C/LDL-C: MDA-LDL-to-LDL cholesterol ratio; MDA-LDL-C/HDL-C: MDA-LDL-to-HDL cholesterol ratio; (MDA-LDL/LDL-C)/HDL-C: (MDA-LDL/LDL-C) to HDL cholesterol ratio.
